# Real-world effectiveness of cytisine, bupropion, and nicotine replacement therapy in a smoking cessation clinic: A single-center retrospective cohort study

**DOI:** 10.18332/tid217961

**Published:** 2026-03-27

**Authors:** Hayriye Bektaş Aksoy

**Affiliations:** 1 Department of Chest Disease, Faculty of Medicine, Giresun University, Giresun, Türkiye

**Keywords:** bupropion, cytisine, nicotine replacement therapy, smoking cessation, treatment adherence

## Abstract

**INTRODUCTION:**

Pharmacological treatments such as cytisine, bupropion, and nicotine replacement therapy (NRT) are widely used to support smoking cessation. However, evidence comparing their effectiveness in real-world clinical settings, particularly within smoking cessation outpatient clinics, remains limited.

**METHODS:**

This retrospective cohort study evaluated smoking cessation outcomes among patients attending a tertiary smoking cessation outpatient clinic, with follow-up assessments conducted at approximately the first, third, and sixth months. The study was conducted between 1 January 2024 and 28 March 2025. Participants received cytisine, bupropion, or NRT in combination with standardized behavioral counseling. Demographic variables, smoking-related characteristics, treatment adherence, adverse effects, and cessation outcomes were assessed. Smoking cessation was defined as self-reported abstinence during follow-up. Adult patients aged ≥18 years who received smoking cessation treatment were included in the study.

**RESULTS:**

A total of 113 patients were included, of whom 67 (59.3%) successfully quit smoking. Completion of pharmacological treatment was significantly higher among patients who quit smoking compared with those who did not (77.6% vs 30.4%, p<0.001). Cytisine use was more frequent among patients who achieved smoking cessation (58.2% vs 39.1%, p=0.046), whereas bupropion use was higher among those who failed to quit smoking (41.3% vs 20.9%, p=0.019). Nicotine replacement therapy use was not significantly associated with cessation outcomes (p=0.983). Among patients who achieved abstinence, relapse occurred in approximately one-third during follow-up. Improvement in dyspnea was reported more frequently among cytisine users.

**CONCLUSIONS:**

In this real-world outpatient setting, cytisine was associated with higher smoking cessation success compared with bupropion and NRT. Treatment adherence emerged as a key determinant of cessation outcomes.

## INTRODUCTION

Tobacco use remains one of the leading preventable causes of morbidity and mortality worldwide, accounting for more than 8 million deaths annually^1^. Despite global reductions in smoking prevalence, nicotine dependence continues to represent a significant public health challenge, particularly in low- and middle-income countries where cessation support and pharmacotherapies are less accessible^1^. In recent decades, the use of pharmacological treatments such as nicotine replacement therapy (NRT), bupropion, and cytisine has become a cornerstone of evidence-based strategies for smoking cessation, often combined with behavioral counseling to maximize abstinence outcomes^2^.

Cytisine, a naturally occurring plant alkaloid extracted from *Laburnum anagyroides*, functions as a partial agonist of the α4β2 nicotinic acetylcholine receptor^3^. By mimicking nicotine’s action on these receptors, cytisine reduces withdrawal symptoms and craving while attenuating nicotine’s reinforcing effects^3,4^. Randomized controlled trials and observational studies have shown that cytisine significantly increases quit rates compared with placebo and demonstrates efficacy comparable to other first-line pharmacotherapies^3,4^. Its affordability and short 25-day treatment regimen make it a promising option, especially in resource-limited health systems. Furthermore, recent real-world studies in Central and Eastern Europe have demonstrated cytisine’s high tolerability and adherence, reinforcing its role as a practical cessation aid^5^. In Turkey, cytisine has recently gained attention following regulatory approval, yet evidence regarding its real-world effectiveness in outpatient cessation settings remains scarce. Understanding its comparative performance against other established pharmacotherapies, such as NRT and bupropion, is therefore crucial.

Bupropion, a monocyclic antidepressant and norepinephrine–dopamine reuptake inhibitor, exerts its smoking cessation effect by modulating dopaminergic activity within the mesolimbic pathway and antagonizing nicotinic acetylcholine receptors^6,7^. These mechanisms alleviate craving and withdrawal symptoms while simultaneously counteracting the post-cessation depressive mood commonly observed in smokers^6^. Evidence from randomized trials indicates that bupropion approximately doubles the odds of long-term abstinence compared with placebo^6,7^. Additionally, studies have shown that bupropion can be safely and effectively combined with long-acting NRT, offering synergistic effects in highly dependent smokers^7,8^. Despite these advantages, its clinical use may be limited by contraindications such as seizure risk, insomnia, or concurrent psychiatric conditions^6^. Therefore, patient selection, adherence monitoring, and behavioral support remain key determinants of treatment success in real-world practice.

NRT remains the most widely utilized pharmacological intervention for smoking cessation globally. By supplying controlled doses of nicotine through transdermal patches, gums, lozenges, inhalers, or nasal sprays, NRT mitigates withdrawal symptoms and facilitates gradual dissociation from smoking behavior^9^. A meta-analysis indicates that NRT approximately doubles the likelihood of quitting compared with placebo^9^. Combination NRT, in which a steady nicotine patch is complemented by a rapid-acting form such as gum or lozenge, has shown superior outcomes over monotherapy and is endorsed as first-line treatment in most clinical guidelines^10,11^. Nonetheless, adherence remains a critical determinant of real-world effectiveness. A systematic review and meta-analysis demonstrated a strong association between adherence to NRT and higher abstinence rates^12^. Yet adherence rates in community-based populations are notably lower than in controlled trials, especially among younger adults and women, suggesting a gap between clinical efficacy and practical effectiveness^12,13^. Furthermore, data from Turkey highlight variable use of NRT in primary care and smoking cessation clinics, with inconsistent follow-up protocols potentially affecting long-term outcomes^14^.

While pharmacotherapies have substantially improved cessation success, their real-world effectiveness depends heavily on accessibility, patient adherence, and structured clinical support^14^. Smoking cessation clinics, by integrating pharmacological and behavioral interventions, represent a key setting for optimizing these outcomes. However, limited national data exist on the comparative performance of cytisine, bupropion, and NRT in such real-world conditions. Therefore, this study aimed to evaluate patients attending a smoking cessation clinic, focusing on demographic characteristics, pharmacotherapy modalities, and cessation outcomes, with a particular emphasis on the relative effectiveness of cytisine, bupropion, and NRT in routine clinical practice.

## METHODS

### Study design and setting

This retrospective cohort study included adult patients who attended a tertiary smoking cessation outpatient clinic in Turkey and were followed at approximately the first, third, and sixth months as part of routine clinical care. Treatment allocation was based on routine clinical practice rather than randomization. The study included adult patients who presented to the clinic between 1 January 2024 and 28 March 2025, for smoking cessation treatment. The clinic operates under the national tobacco control program and offers evidence-based pharmacological and behavioral interventions. As a real-world observational study, treatment allocation was based on routine clinical judgment and patient characteristics rather than randomization.

### Study population

The study population consisted of individuals aged ≥18 years who voluntarily applied to the smoking cessation clinic during the study period with the primary intention to quit smoking. Patients were evaluated through medical history, clinical examination, and counseling before initiation of pharmacotherapy.

### Inclusion and exclusion criteria

Individuals presenting to the Smoking Cessation Clinic with the purpose of quitting smoking were eligible for inclusion in the study. Patients aged <18 years, pregnant individuals, patients with severe cognitive impairment, cases with incomplete or missing medical data (anamnesis or laboratory records) in the electronic health system, and individuals who were mistakenly registered for smoking cessation while attending the clinic for routine pulmonary examination, were excluded from the study.

### Ethics approval

The study was conducted in accordance with the Declaration of Helsinki and was approved by the Giresun Training and Research Hospital Scientific Research Ethics Committee (Approval No: BAEK-312, Date/Decision No: 14.05.2025/09).

### Study groups

Participants were divided into three groups according to the pharmacological therapy prescribed for smoking cessation. Group 1 consisted of patients who received cytisine-based therapy. Group 2 included patients treated with bupropion-based therapy. Group 3 comprised patients who received nicotine replacement therapy, including nicotine patches, gums, or lozenges, either as monotherapy or in combination, in accordance with routine clinical practice. Behavioral counseling was delivered as part of routine clinical practice and was not based on a strictly standardized protocol, although it followed national smoking cessation guidelines.

### Data collection and variables

Demographic data including age, gender, education level (illiterate, primary school, middle school, high school, university), residence (urban, rural), marital status (single, married), and income level (in multiples of the minimum wage: <1, 1–2, 2–3, >3; or no income) were extracted from electronic health records. Daily cigarette consumption was categorized as 1–10, 11–20, 21–40, or >40. Reasons for application to the smoking cessation clinic included financial burden, concern about health, respiratory symptoms causing discomfort, concern about deterioration in self-care, and social pressure. Nicotine dependence was assessed using the Fagerström test for nicotine dependence^15^ and classified as low (0–3), moderate (4–6), or high (7–10). Comorbidities such as heart disease, diabetes mellitus, cerebrovascular disease, cancer, psychiatric disorders, and chronic lung disease were recorded. Pharmacological data included smoking cessation treatment status, treatment type, adverse effects, relapse status, reasons for failure to complete treatment, perceived changes after smoking cessation, and reasons for relapse. Follow-up assessments were conducted at approximately the first, third, and sixth months after treatment initiation through in-person visits or telephone interviews as part of routine clinical follow-up. During these visits, patients were evaluated for smoking status, relapse occurrence, reasons for relapse, and self-reported improvements after cessation. Smoking cessation was defined as self-reported abstinence for at least seven consecutive days before follow-up assessment. Follow-up visits or telephone interviews were conducted as part of routine clinical care, and not all patients attended all scheduled follow-up assessments. Key demographic and clinical variables were considered as potential confounding factors in the analyses.

### Statistical analysis

Data were analyzed using IBM SPSS Statistics version 22 (IBM Corp., Armonk, NY, USA). Continuous variables were expressed as mean ± SD, and categorical variables as frequencies and percentages. Group comparisons were performed using an independent sample t-test for continuous variables and chi-squared tests for categorical variables. A p<0.05 was considered statistically significant, and all analyses were exploratory in nature, given the observational study design. Given the exploratory nature of the study and the limited sample size, multivariable modeling was not performed.

## RESULTS

A total of 113 patients who applied to the Smoking Cessation Outpatient Clinic were included in the study. Of these, 67 patients (59.3%) successfully quit smoking, while 46 patients (40.7%) did not achieve smoking cessation. The mean age was 44.3 ± 17 years in the smoking cessation group and 47.5 ± 14.3 years in the non-cessation group, with no statistically significant difference between groups (p=0.296). Sex distribution was also similar between the two groups; 40.3% of patients in the cessation group were female, and 59.7% were male, compared with 39.1% female and 60.9% male in the non-cessation group (p=0.901).

There were no significant differences between the groups in education level, area of residence, marital status, income level, or the presence of comorbid diseases. Daily cigarette consumption and nicotine dependence levels assessed by the Fagerström test for nicotine dependence were comparable between patients who quit smoking and those who did not. Among patients who quit smoking, 14.9% had low, 43.3% had moderate, and 41.8% had high nicotine dependence, while corresponding proportions among those who did not quit were 13.0%, 39.1%, and 47.8%, respectively (p=0.816) (Table 1).

**Table 1:** Sociodemographic, clinical, and smoking-related characteristics according to smoking cessation status in a retrospective observational study conducted at a tertiary smoking cessation outpatient clinic in Turkey, January 2024–March 2025 (N=113)

*Characteristics*	*Smoking cessation status*		
	*Quit* *n (%)*	*Did not quit* *n (%)*	*p*
**Age** (years), mean ± SD	44.3 ± 17	47.5 ± 14.3	0.296
**Gender**			
Female	27 (40.3)	18 (39.1)	0.901
Male	40 (59.7)	28 (60.9)	
**Education level**			
Illiterate	0 (0)	1 (2.2)	0.326
Primary school	14 (20.9)	14 (30.4)	
Middle school	10 (14.9)	9 (19.6)	
High school	21 (31.3)	13 (28.3)	
University	22 (32.8)	9 (19.6)	
**Residence**			
Rural	16 (23.9)	14 (30.4)	0.438
Urban	51 (76.1)	32 (69.6)	
**Marital status**			
Single	25 (37.3)	14 (30.4)	0.450
Married	42 (62.7)	32 (69.6)	
**Income level** (multiples of minimum wage)			
<1	12 (17.9)	4 (8.7)	0.468
1–2	28 (41.8)	21 (45.7)	
2–3	13 (19.4)	9 (19.6)	
>3	8 (11.9)	4 (8.7)	
No income	6 (9.0)	8 (17.4)	
**Comorbidity**			
Heart disease	20 (29.9)	13 (28.3)	0.855
Diabetes mellitus	8 (11.9)	8 (17.4)	0.414
Cerebrovascular disease	3 (4.5)	2 (4.3)	0.974
Cancer	4 (6.0)	4 (8.7)	0.579
Psychiatric disorder	12 (17.9)	15 (32.6)	0.072
Chronic lung disease	16 (23.9)	13 (28.3)	0.600
**Daily cigarette consumption**			
1–10	5 (7.5)	5 (10.9)	0.932
11–20	29 (43.3)	20 (43.5)	
21–40	28 (41.8)	18 (39.1)	
>40	5 (7.5)	3 (6.5)	
**Reason for application to the smoking cessation clinic**			
Financial burden	21 (31.3)	6 (13.0)	**0.025**
Concern about health	48 (71.6)	23 (50.0)	**0.019**
Respiratory symptoms causing discomfort	42 (62.7)	32 (69.6)	0.450
Concern about deterioration in self-care	26 (38.8)	10 (21.7)	0.056
Social pressure	11 (16.4)	11 (23.9)	0.323
**Fagerström test for nicotine dependence** (score)			
Low (0–3)	10 (14.9)	6 (13.0)	0.816
Moderate (4–6)	29 (43.3)	18 (39.1)	
High (7–10)	28 (41.8)	22 (47.8)	

Group differences were calculated using t-tests for continuous variables and chi-squared tests for categorical variables.

When reasons for applying to the Smoking Cessation Outpatient Clinic were evaluated, patients who reported financial burden as a reason for application were more likely to quit smoking compared with those who did not (31.3% vs 13.0%, p=0.025). Similarly, health concern was reported more frequently among patients who quit smoking than among those who did not (71.6% vs 50.0%, p=0.019). Concern about deterioration in self-care was reported by 38.8% of patients who quit smoking and 21.7% of those who did not, although this difference did not reach statistical significance (p=0.056). No significant differences were observed between groups in the reasons for application, including respiratory symptoms or social pressure (Table 1).

In terms of treatment adherence, completion of smoking cessation therapy was significantly higher among patients who successfully quit smoking compared with those who did not (77.6% vs 30.4%, p<0.001). The frequency of reported adverse effects, including headache, dizziness, nausea/vomiting, insomnia, dry mouth, and other symptoms, did not differ significantly between the two groups. Approximately half of the patients in both groups reported no treatment-related adverse effects (Table 2).

**Table 2:** Treatment adherence, adverse effects, and pharmacological therapy according to smoking cessation status in a retrospective observational study conducted at a tertiary smoking cessation outpatient clinic in Turkey, January 2024–March 2025 (N=113)

	*Smoking cessation status*		
	*Quit* *n (%)*	*Did not quit* *n (%)*	*p*
**Status of smoking cessation treatment**			
Completed	52 (77.6)	14 (30.4)	**<0.001**
Not completed	15 (22.4)	32 (69.6)	
**Adverse effects**			
Headache	3 (4.5)	5 (13.5)	0.103
Dizziness	5 (7.6)	7 (18.9)	0.085
Nausea/vomiting	8 (12.1)	9 (24.3)	0.109
Lightheadedness	5 (7.6)	3 (8.1)	0.923
Insomnia	9 (13.6)	6 (16.2)	0.722
Dry mouth/thirst	13 (19.7)	6 (16.2)	0.662
No adverse effects	31 (47.0)	17 (45.9)	0.920
**Relapse**			
Yes	23 (34.3)		
No	44 (65.7)		
**Bupropion**			
Yes	14 (20.9)	19 (41.3)	**0.019**
No	53 (79.1)	27 (58.7)	
**Cytisine**			
Yes	39 (58.2)	18 (39.1)	**0.046**
No	28 (41.8)	28 (60.9)	
**Nicotine replacement therapy**			
Yes	13 (19.4)	9 (19.6)	0.983
No	54 (80.6)	37 (80.4)	

Group differences were calculated using chi-squared tests for categorical variables.

Evaluation of pharmacological treatment modalities revealed that treatment with bupropion use was more common among patients who did not quit smoking compared with those who quit (41.3% vs 20.9%, p=0.019). In contrast, treatment with cytisine use was significantly higher among patients who successfully quit smoking (58.2% vs 39.1%, p=0.046). No significant difference was observed between the groups regarding the use of nicotine replacement therapy (19.4% vs 19.6%, p=0.983) (Table 2).

No statistically significant differences were found between users and non-users of bupropion, cytisine, or NRT, with respect to nicotine dependence levels, treatment completion rates, reasons for treatment discontinuation, or the frequency of treatment-related adverse effects. Across all pharmacological modalities, the most commonly reported reasons for failure to complete treatment were insufficient reduction in smoking urge (42.9–60.0%) and adverse effects related to therapy (42.9–50.0%). This marked difference highlights treatment completion as a clinically important factor associated with smoking cessation success in routine outpatient practice (Table 3).

**Table 3:** Treatment-related characteristics and perceived outcomes according to pharmacological therapy in a retrospective observational study conducted at a tertiary smoking cessation outpatient clinic in Turkey, January 2024–March 2025 (N=113)

	*Bupropion treatment*			*Cytisine treatment*			*Nicotine replacement therapy*		
	*Yes* *n (%)*	*No* *n (%)*	*p*	*Yes* *n (%)*	*No* *n (%)*	*p*	*Yes* *n (%)*	*No* *n (%)*	*p*
**Fagerström test for nicotine dependence** (score)									
Low (0–3)	2 (6.1)	14 (17.5)	0.189	8 (14)	8 (14.3)	0.656	6 (27.3)	10 (11.0)	0.144
Moderate (4–6)	13 (39.4)	34 (42.5)		26 (45.6)	21 (37.5)		8 (36.4)	39 (42.9)	
High (7–10)	18 (54.5)	32 (40.0)		23 (40.4)	27 (48.2)		8 (36.4)	42 (46.2)	
**Status of smoking cessation treatment**									
Completed	19 (57.6)	47 (58.8)	0.908	35 (61.4)	31 (55.4)	0.514	12 (54.5)	54 (59.3)	0.682
Not completed	14 (42.4)	33 (41.3)		22 (38.6)	25 (44.6)		10 (45.5)	37 (40.7)	
**Reasons for failure to complete treatment**									
Adverse effects	6 (42.9)	16 (48.5)	0.724	11 (50.0)	11 (44.0)	0.681	5 (50.0)	17 (45.9)	0.820
Insufficient reduction in smoking urge	6 (42.9)	17 (51.5)	0.587	11 (50.0)	12 (48.0)	0.891	6 (60.0)	17 (45.9)	0.430
**Medication-related adverse effects**									
Headache	4 (13.8)	4 (5.4)	0.153	2 (3.8)	6 (11.8)	0.133	2 (9.5)	6 (7.3)	0.736
Dizziness	5 (17.2)	7 (9.5)	0.268	6 (11.5)	6 (11.8)	0.971	1 (4.8)	11 (13.4)	0.270
Nausea/vomiting	5 (17.2)	12 (16.2)	0.900	7 (13.5)	10 (19.6)	0.401	5 (23.8)	12 (14.6)	0.312
Lightheadedness	3 (10.3)	5 (6.8)	0.541	4 (7.7)	4 (7.8)	0.977	1 (4.8)	7 (8.5)	0.564
Insomnia	5 (17.2)	10 (13.5)	0.630	7 (13.5)	8 (15.7)	0.749	3 (14.3)	12 (14.6)	0.968
Dry mouth/thirst	4 (13.8)	15 (20.3)	0.446	13 (25.0)	6 (11.8)	0.083	2 (9.5)	17 (20.7)	0.237
No adverse effects	15 (51.7)	33 (44.6)	0.514	23 (44.2)	25 (49.0)	0.626	11 (52.4)	37 (45.1)	0.552
Bitter taste in the mouth	3 (10.3)	7 (9.5)	0.891	3 (5.8)	7 (13.7)	0.173	4 (19.0)	6 (7.3)	0.105
Mood disturbance	1 (3.4)	4 (5.4)	0.678	3 (5.8)	2 (3.9)	0.663	1 (4.8)	4 (4.9)	0.982
**Perceived changes after smoking cessation**									
Improvement/reduction in dyspnea	5 (35.7)	33 (62.3)	0.075	27 (69.2)	11 (39.3)	**0.015**	5 (38.5)	33 (61.1)	0.139
Reduction/improvement in cough and/or sputum	8 (57.1)	37 (69.8)	0.369	27 (69.2)	18 (64.3)	0.671	9 (69.2)	36 (66.7)	0.860
Increased comfort in social settings	10 (71.4)	43 (81.1)	0.427	31 (79.5)	22 (78.6)	0.928	11 (84.6)	42 (77.8)	0.586
Increased self-confidence	9 (64.3)	35 (66.0)	0.902	26 (66.7)	18 (64.3)	0.840	8 (61.5)	36 (66.7)	0.727
Financial gain	9 (64.3)	36 (67.9)	0.797	27 (69.2)	18 (64.3)	0.671	8 (61.5)	37 (68.5)	0.630

Group differences were calculated using chi-squared tests for categorical variables.

Regarding clinical and psychosocial changes after smoking cessation, improvement or reduction in dyspnea was reported more frequently among patients receiving cytisine compared with those not receiving cytisine (p=0.015). Other perceived benefits, including reduced cough and/or sputum production, increased comfort in social settings, improved self-confidence, and financial gain, were reported at similar rates across treatment modalities (Table 3).

Among patients who experienced relapse, no significant differences were observed between pharmacological treatment groups in terms of completion of smoking cessation treatment, timing of relapse, or reasons for resuming smoking. Across all treatment modalities, the most frequently reported reasons for relapses were inability to resist smoking urges, influence of social environment, and exposure to stressful life events. Relapse information was obtained during routine follow-up visits or telephone interviews (Table 4).

**Table 4:** Relapse characteristics according to pharmacological therapy among patients who quit smoking in a retrospective observational study conducted at a tertiary smoking cessation outpatient clinic in Turkey, January 2024–March 2025 (N=67)

	*Bupropion treatment*			*Cytisine treatment*			*Nicotine replacement therapy*		
	*Yes* *n (%)*	*No* *n (%)*	*p*	*Yes* *n (%)*	*No* *n (%)*	*p*	*Yes* *n (%)*	*No* *n (%)*	*p*
**Status of smoking cessation treatment**									
Completed	6 (42.9)	17 (32.1)	0.450	12 (30.8)	11 (39.3)	0.469	5 (38.5)	18 (33.3)	0.727
Not completed	8 (57.1)	36 (67.9)		27 (69.2)	17 (60.7)		8 (61.5)	36 (66.7)	
**Time to relapse after smoking cessation**									
Within the first month	1 (16.7)	7 (41.2)	0.278	5 (41.7)	3 (27.3)	0.271	2 (40.0)	6 (33.3)	0.732
After one month	5 (83.3)	10 (58.8)		7 (58.3)	8 (72.7)		3 (60.0)	12 (66.7)	
**Reasons for relapse**									
Inability to resist the smoking urge	3 (50.0)	18 (88.2)	0.051	11 (91.7)	7 (63.6)	0.104	3 (60.0)	15 (83.3)	0.263
Decreased motivation to quit	3 (50.0)	13 (76.5)	0.266	8 (66.7)	8 (72.7)	0.752	5 (100)	11 (61.1)	0.095
Influence of social environment	5 (83.3)	11 (64.7)	0.394	7 (53.3)	9 (81.8)	0.271	4 (80.0)	12 (66.7)	0.567
Weight gain	0 (0)	2 (11.8)	0.379	2 (16.7)	0 (0)	0.157	0 (0)	2 (11.1)	0.435
Relapse due to stressful life events	5 (83.3)	10 (58.8)	0.278	7 (58.3)	8 (72.7)	0.271	4 (80.0)	11 (61.1)	0.433

Group differences were calculated using chi-squared tests for categorical variables.

## DISCUSSION

The primary finding of this research was that cytisine treatment was associated with the highest smoking cessation success rate, followed by bupropion, while NRT did not show a statistically significant effect on cessation outcomes. These findings are consistent with the growing body of evidence indicating that cytisine is an effective, safe, and affordable pharmacotherapy for nicotine dependence, particularly in low- and middle-income countries where cost and accessibility limit the use of other agents^3^. Importantly, our findings extend this evidence by demonstrating cytisine’s effectiveness under routine clinical conditions rather than controlled trial environments.

The superior cessation rates achieved with cytisine in this study align with findings from randomized controlled trials and network meta-analyses demonstrating higher abstinence rates with cytisine compared with placebo^2,4^. Cytisine’s pharmacological action as a partial agonist at α4β2 nicotinic acetylcholine receptors effectively mitigates withdrawal symptoms while reducing nicotine reward, thereby promoting abstinence^5^. In the present study, a considerable proportion of cytisine users reported discontinuing treatment early due to achieving abstinence by the fifth day of therapy. This rapid onset of effect has also been reported in previous real-world evaluations, suggesting that cytisine’s partial agonist mechanism suppresses early craving, which may contribute to its high adherence and satisfaction rates. Furthermore, improvement in shortness of breath, reported significantly more often among cytisine users in this study, supports the hypothesis that early abstinence yields measurable respiratory benefits within weeks of cessation^16^.

Bupropion also demonstrated a statistically significant relationship with cessation success in our cohort, though its efficacy was lower than that of cytisine. Bupropion’s dual mechanism – dopaminergic and noradrenergic reuptake inhibition combined with non-competitive antagonism of nicotinic receptors – modulates the reward circuitry associated with nicotine withdrawal^6^. The cessation rates observed in this study are consistent with a previous study reporting significantly higher abstinence rates with bupropion compared with placebo^8^. However, comorbid conditions such as diabetes mellitus and chronic pulmonary disease were more prevalent in the bupropion group, possibly reflecting physician selection bias favoring bupropion in patients with contraindications to NRT or cytisine. Additionally, while bupropion is well tolerated, adverse effects such as insomnia and dry mouth may limit adherence^7^. Nonetheless, its positive impact on mood and anhedonia remains clinically relevant, particularly among smokers with depressive symptoms.

In contrast, NRT did not show a significant association with smoking cessation in this sample. Although meta-analyses consistently demonstrate that NRT significantly increases quit rates compared with placebo^9^, real-world adherence remains suboptimal. Studies have shown that full adherence to NRT regimens is associated with substantially higher cessation success rates^9,12^, while inconsistent use markedly reduces effectiveness. The relatively lower success rate of NRT in our cohort likely reflects limited adherence and underdosing, as many patients reported early discontinuation or sporadic use. Moreover, behavioral support accompanying NRT is crucial for optimizing outcomes^10^; in resource-limited clinical settings, such structured follow-up may be inconsistent, contributing to lower observed efficacy.

Treatment adherence emerged as one of the most critical determinants of cessation success in this study, with patients completing their pharmacotherapy achieving more than twice the quit rate of those who discontinued prematurely. This finding aligns with previous work emphasizing adherence as a central factor in treatment success across all pharmacotherapies^12,17^. The strong association between adherence and cessation underscores the importance of structured behavioral counseling and follow-up in smoking cessation clinics. In Turkey, recent analyses of national cessation programs have highlighted that patient follow-up intensity and clinician continuity are key predictors of success^18^.

Beyond pharmacological efficacy, motivational and socioeconomic factors strongly influenced cessation outcomes. In our study, patients who reported health concerns or the financial burden of smoking as reasons for attending the clinic were significantly more likely to achieve abstinence^19^. These findings are consistent with recent population-based evidence. A large study from England found that health concerns remain the leading reason for quit attempts, cited in approximately half of cases between 2018 and 2023. In contrast, financial reasons have become increasingly salient, motivating over one-quarter of recent quit attempts^19^. Likewise, a multi-country analysis across low- and middle-income nations found that socio-economic status and affordability strongly determine cessation behaviors, with higher quit rates among those experiencing economic constraints or unemployment^13^. At the macroeconomic level, tobacco use imposes a substantial financial burden worldwide due to healthcare costs and productivity losses^20^. Together, these data indicate that both perceived health risks and financial pressures act as powerful, complementary motivators for cessation in diverse populations.

The low overall incidence of side effects across treatment groups in this study supports the established safety profiles of cytisine, bupropion, and NRT^2^. The most common adverse events – dry mouth, nausea, and insomnia – were mild and self-limited, consistent with prior reports^6,16^. Importantly, no serious neuropsychiatric events were observed, which supports the broader safety of these agents in real-world clinical practice. Functional improvements were reported across all groups, suggesting that early perceived health benefits may reinforce motivation to maintain abstinence.

### Strengths and limitations

This study has several limitations that should be acknowledged. First, its retrospective and single-center design limits the generalizability of the findings and precludes causal inference. Second, smoking cessation outcomes were self-reported and not confirmed biochemically (e.g. by exhaled carbon monoxide or cotinine levels), which may have led to information bias and misclassification, potentially resulting in an overestimation of abstinence rates. Third, the intensity and quality of behavioral counseling could not be standardized or quantitatively assessed, which could affect treatment adherence and cessation success. Fourth, socio-economic and psychosocial variables, such as education level, motivation, or household smoking exposure, were not comprehensively documented in the clinical records. Additionally, because multivariable adjustment for potential confounding factors was not performed, residual confounding cannot be excluded. Finally, the sample size within each pharmacotherapy group, particularly among NRT users, was modest, which may have limited the power to detect smaller between-group differences.

Despite these limitations, the study provides valuable real-world insights into the comparative effectiveness of cytisine, bupropion, and NRT in routine clinical practice within a tertiary healthcare setting. Nevertheless, the real-world design of the study enhances its external validity and provides clinically relevant insights into routine smoking cessation practice.

## CONCLUSIONS

In this real-world study of patients attending a tertiary smoking cessation clinic, cytisine demonstrated the highest rate of smoking cessation success, followed by bupropion. At the same time, NRT showed no significant association with abstinence outcomes. These results reinforce the evidence supporting cytisine as an effective, safe, and affordable pharmacological option for tobacco dependence treatment. Bupropion remains a valuable alternative, particularly for smokers with comorbid depressive symptoms, whereas the limited impact of NRT highlights the importance of adherence and structured behavioral support.

Treatment completion emerged as the strongest determinant of success, underscoring the necessity of close clinical follow-up and patient-centered counseling in smoking cessation programs. Future multicenter, prospective studies using biochemical verification of abstinence and standardized behavioral interventions are warranted to validate and extend these real-world findings.

## Data Availability

The data supporting this research are available from the author on reasonable request.
